# Preparation of ZnGa_2_O_4_ nanoflowers and their full-color luminescence properties

**DOI:** 10.1038/s41598-023-41658-5

**Published:** 2023-09-02

**Authors:** Yan Liu, Tingting Zheng, Xiuyun Zhang, Chen Chen

**Affiliations:** 1 Department of Pharmacy, Shandong University of Traditional Chinese Medicine, Jinan, 250355 Shandong China; 2 Key Laboratory of New Material Research Institute, Department of Acupuncture-Moxibustion and Tuina, Shandong University of Traditional Chinese Medicine, Jinan, 250355 China

**Keywords:** Materials for optics, Nanoscale materials

## Abstract

Gallate material, a luminescent matrix with excellent performance is normally prepared by vapor deposition or solid phase sintering method at high temperature. However, it has not been solved to prepare gallate-based fluorescent materials with full-color luminescent properties at low temperature. In this paper, ZnGa_2_O_4_ undoped or doped with Cr or Mn nanoflowers composed of nanosheet-level structure were prepared by hydrothermal method at low temperature. Under ultraviolet light irradiation, ZnGa_2_O_4_, ZnGa_2_O_4_:Mn^2+^ and ZnGa_2_O_4_:Cr^3+^ display three primary colors of blue, green and red luminescence through self-excitation, Mn^2+^ and Cr^3+^ excitation respectively. The solid fluorescence yields of blue, green, and red colors are 32.3, 36.5, and 40.7%, respectively. It is highly expected to be applied to color display, biological imaging, white light devices.

## Introduction

In recent years, inorganic luminescent materials have attracted people's attention because of their wide applications in fluorescence imaging, color display, white Light Emitting Diodes (LEDs) and so on^1–3^. Therefore, constructing and designing efficient inorganic luminescent materials has become a hot topic for material scientists. Zinc gallate (ZnGa_2_O_4_), a ternary spinel material with a band gap of 4.4–4.7 eV, has exhibited excellent potential in future display system due to its prominent blue emission, high chemical and thermal stability, and good cathodoluminescence characteristics at low-voltage^4,5^. Compared with other luminescent materials, zinc gallate can be self-excited by Ga-O group, and has blue light emission^6,7^. ZnGa_2_O_4_ can also be used as the matrix of fluorescent materials, which has high luminous efficiency and narrow spectral band. The luminescent color can be changed by adjusting the surface properties^4,8^ and composition of fluorescent materials^9,10^, or by doping the dopant activators^11,12^. Most fluorescent materials use rare earth metals as activators, such as Eu^3+^, Tb^3+^, Y^3+^, etc.^13–15^, but rare earth metals are expensive and lack of resources. Previous studies have shown that transition metal ions Mn^2+^ and Cr^3+^ can be used as activators of fluorescent materials, such as ZnGa_2_O_4_:Mn^2+^ emitting green fluorescence and ZnGa_2_O_4_:Cr^3+^ emitting red fluorescence^11,16,17^. Various synthetic methods have been adopted to synthesize these fluorescent materials, such as thermal evaporation^18^, solid-state reaction method^19^, Chemical Vapor Deposition (CVD), Atomic Layer Deposition (ALD) and so on^20^. These synthetic methods usually require high reaction temperature, which is over 900 °C, so the energy consumption is high, which brings hidden dangers to environmental pollution control. Therefore, it is an urgent problem to find a low-temperature synthesis method for energy saving and emission reduction in the field of synthesis of full-color luminescent materials.

In this paper, undoped (ZnGa_2_O_4_), Mn^2+^-doped (ZnGa_2_O_4_:Mn^2+^) and Cr^3+^-doped (ZnGa_2_O_4_:Cr^3+^) nano-luminescent materials have been synthesized at low temperature by one step hydrothermal method under the template action of ethylenediamine. These luminescent materials are composed of 5 μm-sized nano-flowers, each of which is composed of 6–10 nm nanoflake hierarchical structure. Under ultraviolet irradiation, ZnGa_2_O_4_, ZnGa_2_O_4_:Mn^2+^ and ZnGa_2_O_4_:Cr^3+^ display three primary colors of blue, green and red through self-excitation, Mn^2+^ excitation and Cr^3+^ ion excitation, respectively.

## Methods

### Raw materials and reagents

Gallium nitrate hydrate (Ga(NO_3_)_3_·xH_2_O), Zinc acetate dihydrate (Zn(CH_3_COO)_2_·2H_2_O), Manganese acetate tetrahydrate (Mn(CH_3_COO)_2_·4H_2_O), Chromium nitrate nonahydrate (Cr(NO_3_)_3_·9H_2_O), and Anhydrous ethylenediamine (NH_2_(CH_2_)_2_NH_2_) are analytically pure and boughten from Sigma-Aldrich Shanghai trading Co., Ltd.. Anhydrous ethanol (CH_3_CH_2_OH), ≥ 99.9% are boughten from Shanghai Sinopharm Chemical Co., Ltd. All reagents were not further treated before use. Water used in the experiment is Milli-Q ultrapure water.

### Sample preparation

#### Synthesis of ZnGa_2_O_4_

Ga(NO_3_)_3_·xH_2_O 0.512 g (2 mmol) and Zn(CH_3_COO)_2_·2H_2_O 0.220 g (1 mmol) are added into 20 mL deionized water, magnetically stirred for 20 min at room temperature. And then 10 mL anhydrous ethylenediamine is added into the above solution, with continue stirring for 20 min. The mixed solution is transferred to a 50 mL reaction kettle and placed into an oven, and stirred at 220 °C for 12 h. The white precipitate was obtained by washed with water and absolute ethanol several times, and dried at 60 °C for 12 h.

#### Synthesis of ZnGa_2_O_4_:Mn^2+^

Ga(NO_3_)_3_·xH_2_O 0.512 g (2 mmol) and Zn(CH_3_COO)_2_·2H_2_O 0.220 g (1 mmol) are added into 17.5 mL deionized water, magnetically stirred for 20 min at room temperature. And then 2.5 mL Mn(CH_3_COO)_2_ solution with a concentration of 4 mmol L^−1^ is added to above solution so that the concentration of Mn^2+^ is 1% of that of Zn^2+^, and magnetically stirred at room temperature for 20 min. Next, 10 mL anhydrous ethylenediamine is added into the above solution, with continue stirring for 20 min. The mixed solution is transferred to a 50 mL reaction kettle and placed into an oven, and stirred at 220 °C for 12 h. The white precipitate was obtained by washed with water and absolute ethanol several times, and dried at 60 °C for 12 h.

#### Synthesis of ZnGa_2_O_4_:Cr^3+^

The method is as same as the preparation of ZnGa_2_O_4_:Mn^2+^, and finally the concentration of Cr^3+^ is 0.5% of that of Zn^2+^.

### Sample characterization

The phase structure of the sample was determined at room temperature by X-ray powder diffraction analyzer (Rigaku D/Max 2200PC, graphite monochromator filter, Cu Kα radiation, λ = 0.1542 nm) with the condition of tube voltage 40 kV, the tube current 20 mA, the scanning range 10°–80° (2θ) and the scanning speed 10 min^−1^. The morphology and microstructure of the product were characterized by transmission electron microscope (JEM-100CXII, accelerating voltage 80 kV), high resolution transmission electron microscope (Philips Tecnai 20U-TWIN, accelerating voltage 200 kV) and scanning electron microscope (FE-SEM, S-4800, Hitachi, accelerating voltage 5 kV). X-ray photoelectron spectrometer (PHI-5300 ESCA spectrometer, Perkin Elmer, Al Kα as excitation light source) was used to analyze the surface properties of the samples. Before the spectrogram analysis, the electron binding energies of all elements were corrected with the C_*1s*_ peak at 284.6 eV as reference. Photoluminescence (PL) and fluorescence lifetime of samples were measured by Agilent Cary Eclipse Fluorescence Spectrometer. The UV–Vis absorption spectrum of the sample at room temperature was tested by Agilent Cary Series UV-VIS spectrometer, and BaSO_4_ was used as baseline correction before the test. The infrared spectrum of the sample was tested by NICOLET FT-IR spectrometer, and the KBr was used as the background.

## Results and discussion

### Phase structure and morphology characteristics

**Figure 1:**
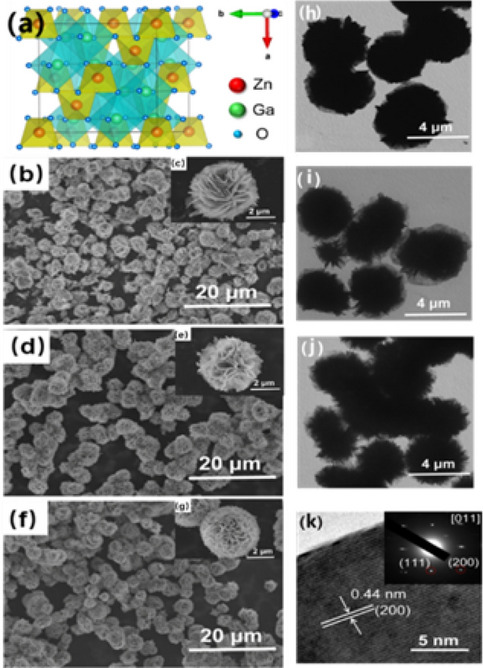
(**a**) Cubic spinel structure of ZnGa_2_O_4_, SEM images of (**b**-**c**) ZnGa_2_O_4_, (**d**–**e**) ZnGa_2_O_4_:Mn^2+^ and (**f**–**g**) ZnGa_2_O_4_:Cr^3+^, TEM images of (**h**) ZnGa_2_O_4_, (**i**) ZnGa_2_O_4_:Mn^2+^, (**j**) ZnGa_2_O_4_:Cr^3+^ (**k**) HRTEM image of ZnGa_2_O_4_, with the inset showing the corresponding SAED patterns.

ZnGa_2_O_4_ is a bimetallic oxide composed of ZnO and Ga_2_O_3_, with *Fd-3m* space group symmetry, a = b = c = 8.335, and spinel structure with chemical formula AB_2_O_4_, in which Zn^2+^ occupying tetrahedral center, Ga^3+^ occupying octahedral center^21^, as shown in Fig. 1a. Usually, the charge imbalance caused by the introduction of impurity ions is unfavorable to the luminous intensity of luminophores, so higher energy is needed to eliminate the charge imbalance, such as calcination at high temperature for a long time^22^. The effective compensation factor φ when ions are substituted was calculated according to the formula *φ* = *Z/r*, in which *Z* is the charge number of ions and *r* is the effective radius of ions^23^. The greater the difference of *φ*, the more difficult it is to substitute ions. As shown in Table S1, for hexa-coordinate substitution of Mn^2+^, the effective compensation factor* φ* is 2.41, which is much lower than that of Ga^3+^ 4.83, so it is difficult for Mn^2+^ to replace Ga^3+^, while easy to replace Zn^2+^ due to the small difference of effective compensation factor^11,17^. Furthermore, in the substitution reaction of ZnGa_2_O_4_, ions with similar effective radii are easy to be substituted with each other, that is, Mn^2+^ replaces Zn^2+^ to produce four coordinate substitutions, and Cr^3+^ replaces Ga^3+^ to produce six coordinate substitution^17,24^.

The SEM photos of the three nanomaterials (ZnGa_2_O_4_, ZnGa_2_O_4_:Mn^2+^ and ZnGa_2_O_4_:Cr^3+^) with different magnification is shown in Fig. 1b–g. They all present a flower-like nanostructure of about 5 μm, which is composed of multiple nanosheet-level substructures with a thickness of 6–10 nm interspersed together. The difference between the three samples is that the nanosheets composed of ZnGa_2_O_4_ are thicker and the degree of curling of the nanosheets is smaller, while the nanosheets doped with Mn^2+^ and Cr^3+^ are thinner, and the nanosheets are freely curled to form spherical nanoflowers. As shown in Fig. S1. Zn, Ga, O and the corresponding doped elements Mn and Cr are uniformly distributed in the flower-like nanostructures confirmed by the element distribution surface scans. The flower-like structure of ZnGa_2_O_4_, ZnGa_2_O_4_:Mn^2+^ and ZnGa_2_O_4_:Cr^3+^ can also be seen from the TEM photos in Fig. 1h–j. Each flower is composed of the sub-structure of nano-flakes, and the size of nano-flowers is about 5 μm. From the contrast of the electron microscope photos, it can be clearly seen that the nano-flakes constituting the flower-like structure become thinner in turn, which is similar to that of Scanning electron microscope (SEM). The phenomenon may be due to the impurity ions adsorbed on the initial grain surface during hydrothermal process, which inhibiting the crystallization of the material to some extent, preventing the grain growth in some directions, and resulting in the formation of thinner nanosheets. High-resolution photos and corresponding selective electron diffraction photos of ZnGa_2_O_4_ are shown in Fig. 1k. The lattice spacing 0.44 nm is corresponding to the (200) crystal plane of ZnGa_2_O_4_, and the selective electron diffraction photo clearly shows single crystal structure of a ZnGa_2_O_4_ nanoflake. In the single crystal structure of the sheet, the diffraction points correspond to (200) and (111) crystal plane. The direction of the crystal zone axis is confirmed to [011] by calculating, therefore the exposed surface of the nanoplate is (110) plane.

**Figure 2:**
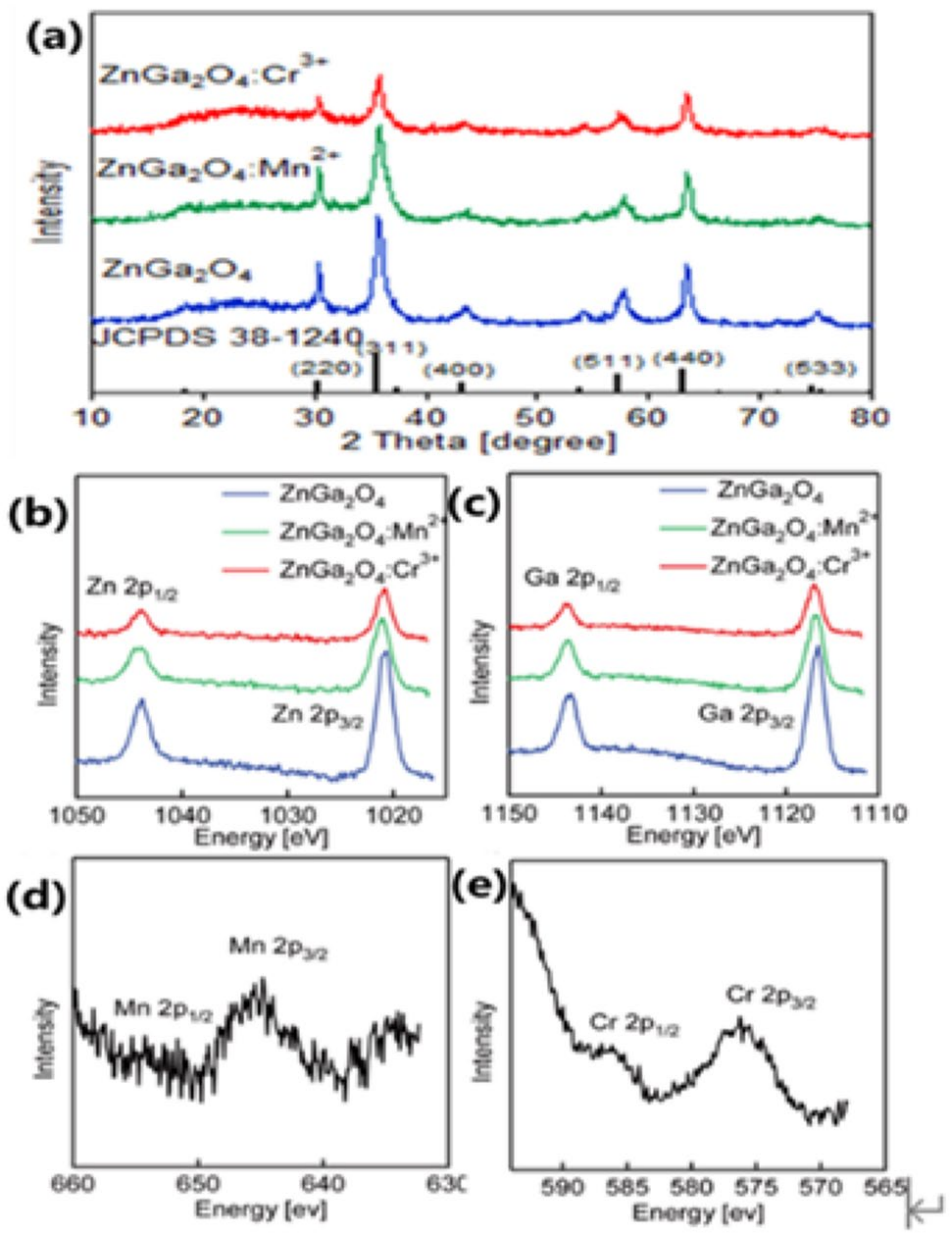
(**a**) XRD patterns of ZnGa_2_O_4_, ZnGa_2_O_4_:Mn^2+^ and ZnGa_2_O_4_:Cr^3+^. The XPS spectra of ZnGa_2_O_4_, ZnGa_2_O_4_:Mn^2+^ and ZnGa_2_O_4_: Cr^3+^: (**b**) Zn 2p, (**c**) Ga 2p, (d) Mn 2p in ZnGa_2_O_4_:Mn^2+^ and (**e**) Cr 2p in ZnGa_2_O_4_:Cr^3+^.

As shown in Fig. 2a the diffraction peaks of ZnGa_2_O_4_, ZnGa_2_O_4_:Mn^2+^ and ZnGa_2_O_4_:Cr^3+^ all correspond to the diffraction peaks of standard card JCPDS38-1240 ZnGa_2_O_4_, which are cubic spinel structure. After doping Mn^2+^ and Cr^3+^, the intensity of the diffraction peak decreases, and the corresponding FWHM (full width at half maximum) increases in sequence. The results of X-ray diffractometer (XRD) analysis also indicate that the secondary structure nanosheets that make up the nanoflowers become thinner in turn after doping Mn^2+^ and Cr^3+^, which consistent precisely with the observation results of SEM and transmission electron microscopy (TEM). As shown in Fig. 2b–e the electron binding energies of 2p_1/2_ orbitals and 2p_3/2_ orbitals of Ga are located at 1143.6 eV and 1116.8 eV, respectively. And the electron binding energies of 2p_1/2_ orbitals and 2p_3/2_ orbitals of Zn are located at 1043.8 eV and 1021.1 eV, respectively. The characteristic peaks of electron binding energies located at 654.4 eV and 645.5 eV belong to 2p_1/2_ orbitals and 2p_3/2_ orbitals of Mn, respectively and manganese ions show +2 valences. The characteristic peaks of electron binding energy at 586.5 eV and 576.5 eV respectively belong to 2p_1/2_ orbitals and 2p_3/2_ orbitals of Cr and chromium ions show +3 valences. The intensity of these characteristic peaks is small due to the low contents of Mn and Cr.

### Infrared and ultraviolet spectral characteristics

In order to understand the chemical composition of the sample, the Fourier transform infrared spectrum is conducted. The Fourier Transform infrared spectroscopy (FT-IR) of ZnGa_2_O_4_, ZnGa_2_O_4_:Mn^2+^ and ZnGa_2_O_4_:Cr^3+^ in Fig. S2 also indicate the samples were binary metal oxides consisting of Zn-O and Ga-O groups. As showed in Fig. S2, the broad absorption peak at the 3445 cm^−1^ wavelength belongs to the stretching vibration of O-H and N-H. The stretching vibration of N-H may come from the residual ethylenediamine in the sample, but there is no obvious stretching vibration peak of it near 1190 cm^−1^. Therefore, the residual ethylenediamine may be very small and can be ignored after repeated cleaning of water and anhydrous ethanol. In the fingerprint region at low wavelength, the larger peaks of 585 cm^−1^ and 425 cm^−1^ are attributed to the vibration absorption of Zn-O and Ga-O, respectively. Through the infrared spectrum analysis, no other vibration peaks are observed except Zn-O and Ga-O in the sample, so it is determined that the sample is a binary metal oxide composed of Zn-O group and Ga-O group. The UV-vis absorption spectra of ZnGa_2_O_4_, ZnGa_2_O_4_:Mn^2+^ and ZnGa_2_O_4_:Cr^3+^ samples are shown in Fig. S3. It can be seen from the figure that the absorption regions of the three samples are basically the same, and there is only absorption in the region smaller than 350 nm. It is also confirmed that ZnGa_2_O_4_ can only be excited at wavelengths less than 350 nm. However, in the 300–350 nm wavelength region, compared with the absorption peak of ZnGa_2_O_4_, the absorption of ZnGa_2_O_4_:Mn^2+^ and ZnGa_2_O_4_:Cr^3+^ increases slightly, which may be due to the absorption of a small amount of Mn and Cr itself, because the amount of Mn^2+^ and Cr^3+^ is very small, only 0.4% and 1%, so the absorption of these two elements is also very weak.

### Luminescent properties

**Figure 3:**
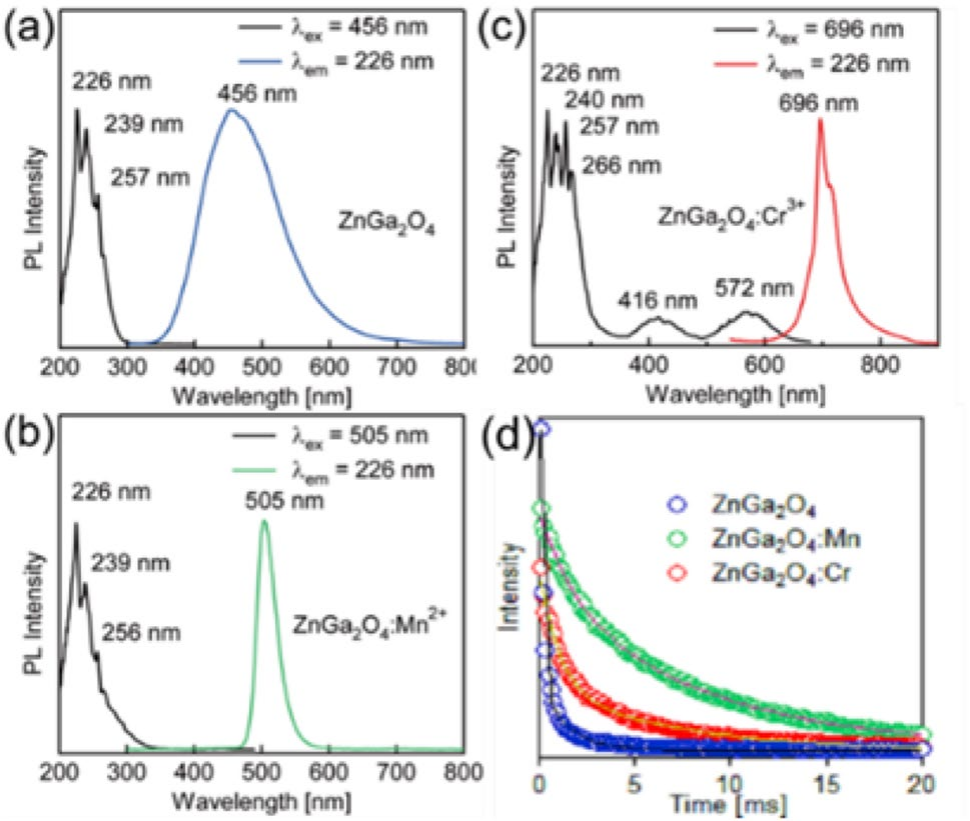
Excitation and emission spectra of (**a**) ZnGa_2_O_4_, (**b**) ZnGa_2_O_4_:Mn^2+^, (**c**) ZnGa_2_O_4_:Cr^3+^ and (**d**) photoluminescence decay curves and bi-exponential fittings for the ZnGa_2_O_4_, ZnGa_2_O_4_:Mn^2+^ and ZnGa_2_O_4_: Cr^3+^.

The excitation and emission spectra of ZnGa_2_O_4_, ZnGa_2_O_4_:Mn^2+^ and ZnGa_2_O_4_:Cr^3+^ are shown in Fig. 3a–c. For undoped ZnGa_2_O_4_, three peaks can be seen in the excitation spectrum, which are located at 226 nm 239 nm and 257 nm, respectively. These excitation peaks are caused by the charge transfer from O^2−^ to octahedral center Ga^3+^ and the ultraviolet absorption of ZnGa_2_O_4_ itself^25^. The emission spectrum obtained using 226 nm as the excitation wavelength is a broad peak with the highest peak of 456 nm in the range of 340–750 nm. This broad emission peak is in all probability caused by the self-excitation of Ga-O hexahedron in the spinel structure. The luminescent properties of fluorescent host materials are usually changed by introducing impurity ions^26^, that is also applicable to ZnGa_2_O_4_ host materials. Mn^2+^-doped ZnGa_2_O_4_ has green fluorescence emission, as shown in Fig. 3b. Except for the charge transfer from O^2-^ to Ga^3+^ in the octahedral center and the ultraviolet absorption of ZnGa_2_O_4_ itself, the absorption of Mn^2+^ excites a red shift of 32 near 300 nm, which is consistent with the analysis of ultraviolet-visible absorption spectrum in Fig. S3. Due to the activation of Mn^2+^, the emission spectrum with the highest emission peak of 505 nm is located in the range of 470–600 nm with the excitation wavelength of 226 nm.

After enlarged locally as shown in Fig. S4, the five smaller excitation peaks located between 351–443 nm and centered at 351 nm, 379 nm, 410 nm, 422 nm and 443 nm respectively correspond to ^5^A_1_–^4^E, ^6^A_1_–^4^T_2_, ^6^A_1_–^4^A_1_, ^4^E_1_ and ^6^A_1_–_4_T of Mn^2+^.^27^ When excited at 226 nm, ZnGa_2_O_4_:Mn^2+^ has green emission at 505 nm, which belongs to the ^4^T_1_–^6^A_1_ d orbital electron forbidden transition of Mn^2+^^11,17^. This is the process of energy transfer from ZnGa_2_O_4_ matrix to Mn^2+^^27^. The transition process of ^4^T_1_–^6^A_1_ of Mn^2+^ is accompanied by strong 3d shell lattice vibration coupling, and is affected by crystal field and symmetric sites. If Mn^2+^ is in a weak crystal field, i.e. tetrahedron, the splitting of excitation energy will be weak, which will be accompanied by high energy emission, that is, green light and if Mn^2+^ is in a strong crystal field, i.e., octahedron, it will emit yellow or red light^17,27^, which is consistent with our previous analysis of crystal structure. In our investigation, Mn^2+^ replaces Zn^2+^ with similar ionic radius in cubic ZnGa_2_O_4_ matrix to generate tetrahedral coordination and emit green light, which is completely consistent with the test results of fluorescence spectrum.

The excitation and emission spectra of ZnGa_2_O_4_:Cr^3+^ are shown in Fig. 3c. There is a wide excitation peak between 200–350 nm, including four intensity excitation peaks (226 nm, 240 nm, 257 nm, 266 nm), which related to the charge transfer transition of O^2−^ to the octahedral center Ga^3+^ and the absorption transition with the belt, and the excitation spectrum of 300–350 nm is caused by the absorption of Cr^3+^. A red emission peak at 696 nm was obtained by excitation at 226 nm, which was attributed to the ^2^E–^4^A_2_ characteristic transformation of Cr^3+^^28^. Meanwhile, a similar red emission peak at 696 nm was obtained by excitation at 416 nm and 572 nm. These two excitation peaks at 416 nm and 572 nm are caused by d–d electron-electron transitions of Cr^3+^^24,29^, corresponding to the ^4^A_2_–^4^T_1_ and ^4^A_2_–^4^T_2_ characteristic transitions of Cr^3+^, respectively^30^.

Undoped, Mn^2+^ doped and Cr^3+^ doped ZnGa_2_O_4_ have blue, green and red emission properties under ultraviolet (UV) excitation, respectively. The excitation wavelengths used in the emission spectra of ZnGa_2_O_4_, ZnGa_2_O_4_:Mn^2+^ and ZnGa_2_O_4_:Cr^3+^ are all 226 nm, indicating that the hetero ions in ZnGa_2_O_4_:Mn^2+^ and ZnGa_2_O_4_:Cr^3+^ can effectively enter the lattice of ZnGa_2_O_4_ under hydrothermal conditions to replace Zn^2+^ and Ga^3+^ to form tetrahedral and octahedral coordination, respectively. This is related to the addition of an appropriate amount of ethylenediamine during hydrothermal. Ethylenediamine aqueous solution is a strongly alkaline solution, which plays an effective role in promoting the crystallization of materials and the entry of hetero ions into the crystal lattice of the matrix under the condition of hydrothermal high temperature and high pressure. We also conduct experiment keeping other conditions remaining the same without ethylenediamine in the synthesis. The obtained ZnGa_2_O_4_ doped with Mn^2+^ and Cr^3+^ does not show green and red emission properties after UV excitation, indicating that it is difficult for hydrothermal hetero ions to enter into the lattice of ZnGa_2_O_4_ matrix under the condition of non-strong alkaline solvent.

The fluorescence attenuation curves of ZnGa_2_O_4_, ZnGa_2_O_4_:Mn^2+^ and ZnGa_2_O_4_:Cr^3+^ are fitted exponentially as shown in Fig. 3d. The attenuation curves of the three samples are all fitted by double exponents, which are in accordance with the formula.

1 $$I = I_{1} {\text{exp}}\left( { - t/\tau_{1} } \right) + I_{2} {\text{exp}}\left( { - t/\tau_{2} } \right)$$

where *I* is the fluorescence intensity when the time is *t*, *I*_*1*_ and *I*_*2*_ are fitting constants, and *τ*_*1*_ and *τ*_*2*_ are fluorescence lifetime. After fitting, each sample corresponds to two millisecond lifetimes, a shorter lifetime *τ*_*1*_ and a relatively longer life *τ*_*2*_. The specific fitting parameters are shown in Table S2. The longer lifetimes of each sample of ZnGa_2_O_4_, ZnGa_2_O_4_:Mn_2+_ and ZnGa_2_O_4_:Cr^3+^ correspond to the self-excitation of Ga-O in the bulk phase of luminescent materials, the ^4^T_1_-^6^A_1_ transition of Mn^2+^ and the ^2^E–^4^A_2_ transition of Cr^3+^, respectively. The short lifetime of the three samples is due to the fact that the surface effect of the materials has a great influence on the luminescence lifetime. These materials are all composed of ultra-thin 6–10 nm nanosheets with large surface area, and the increase of surface atoms leads to the appearance of more activated ions on the surface of the nanosheets. However, the impurities, unsaturated bonds, vacancies and other surface defects on the surface of the nanoparticles will quenched the activated ions and lead to radiation-free transition, thus shortening the life of the activated ions.^8^

**Figure 4:**
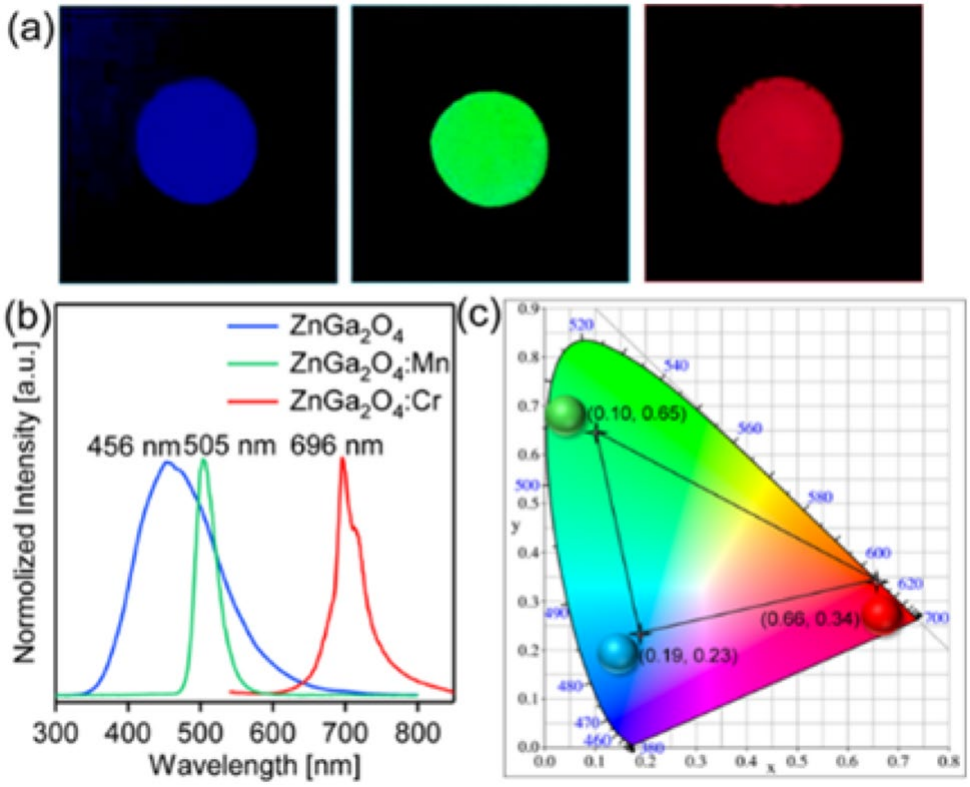
(**a**) Digital images of ZnGa_2_O_4_, ZnGa_2_O_4_:Mn^2+^, and ZnGa_2_O_4_:Cr^3+^ on glass substrates under UV light excitation. (**b**) Normalized PL emission spectra of ZnGa_2_O_4_, ZnGa_2_O_4_:Mn^2+^, and ZnGa_2_O_4_:Cr^3+^ samples. (**c**) The for ZnGa_2_O_4_ (0.19, 0.23), ZnGa_2_O_4_:Mn^2+^ (0.10, 0.65), and ZnGa_2_O_4_:Cr^3+^ (0.66, 0.34) samples.

Excited by 254 nm's handheld UV lamp, ZnGa_2_O_4_, ZnGa_2_O_4_:Mn^2+^ and ZnGa_2_O_4_:Cr^3+^ appear bright blue, green and red, respectively. Their optical photos are shown in Fig. 4a. The solid fluorescence yields of blue, green, and red colors are 32.3, 36.5, and 40.7%, respectively. Under the light excitation of 226 nm wavelength, the Normalized fluorescence emission spectra of the three materials are shown in Fig. 4b. The maximum fluorescence emission spectra of the three materials are located in 456 nm, 505 nm and 696 nm, respectively, which basically correspond to the central regions of blue, green and red. Their emission spectra are imported into the CIE color coordinate software, respectively, and the color coordinate diagram shown in Fig. 4c is obtained. The color coordinates are located at (0.19, 0.23), (0.10, 0.65) and (0.66, 0.34), respectively, which indicates that any color including white in the triangular area connected by the three points can be obtained by changing the ratio of the three luminescent materials.

**Figure 5:**
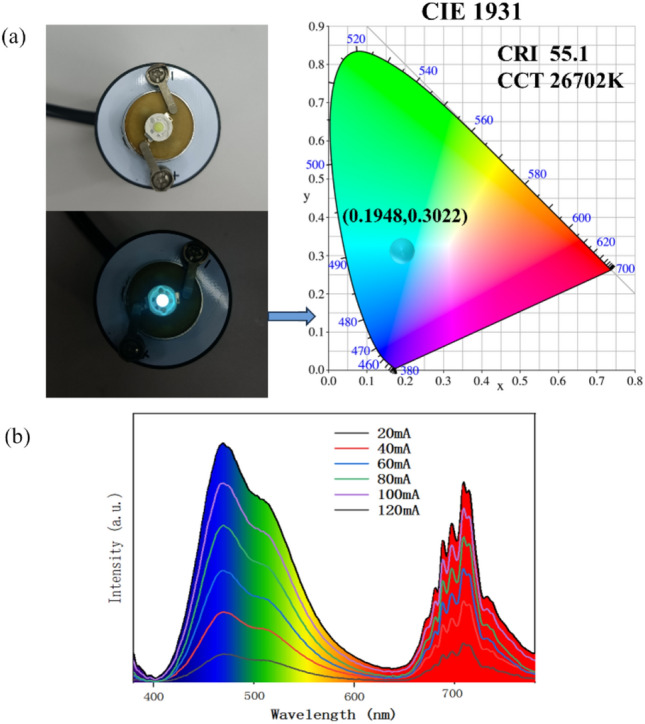
(**a**) Encapsulated white LED devices and emitting colors. (**b**) The relationship between the luminous intensity of packaged white LED and the input forward current.

In order to demonstrate whether there is white luminescence phenomenon when mixing the three samples, we conducted packaged white LED luminescence testing on their mixture. As shown in Fig. 5a, the encapsulated white LED device and the emitting color are shown. The encapsulated white LED prepared has a correlated colour temperature (CCT) of 26702K and a color rendering index (CRI) of 55.1. Within the constant voltage range of 20 mA to 120 mA and 3 V, the relationship between the luminous intensity of the encapsulated white LED and the input forward current is shown in Fig. 5b. From the color of the encapsulated white LED device and the CIE chromaticity diagram, we can see that the light emitted by this mixture is not pure white light, but a cyanish white-like light. According to the emission spectrum of the mixture, we speculate that the reason is that the emission wavelength of red light is mostly in the invisible near-infrared region after 650 nm, and there is an empty window in the wavelength range of 600–650 nm, resulting in the emission color of encapsulated white LED being a cyan white light, rather than a pure white light like the results made by other phosphors^31,32^.

## Conclusions

ZnGa_2_O_4_ nanoflowers with single size composed of ZnGa_2_O_4_ flake substructures with a thickness of 6-10 nm were synthesized by a simple hydrothermal method under the action of ethylenediamine template. The luminescence color of ZnGa_2_O_4_ is controlled by doping Mn^2+^ and Cr^3+^. Under UV excitation, undoped ZnGa_2_O_4_, ZnGa_2_O_4_ doped with Mn^2+^ and Cr^3+^ have blue, green and red emission properties, respectively. The luminescence properties of the same matrix material with three primary colors are obtained. The blue, green and red fluorescence comes from the self-excited electron transfer of Ga-O itself and the 3d electron energy transfer of Mn^2+^ and Cr^3+^. The three samples are mixed to do the encapsulated white LED luminescence test, which has the phenomenon of cyan white luminescence. These ZnGa_2_O_4_-based fluorescent nanomaterials are expected to be used in color display, biological imaging and white light devices.

### Supplementary Information

Supplementary Information.

## Data Availability

All data generated or analyzed during this study are included in this published article (and its Supplementary Information files).
