# Supplementary material for: Preparation of ZnGa2O4 nanoflowers and their full-color luminescence properties
Source: Sci Rep. 2023 Sep 2;13:14430. doi: 10.1038/s41598-023-41658-5 (PMC10475037; doi:10.1038/s41598-023-41658-5)
Supplement: Supplementary file 1 — Supplementary Information. [file 41598_2023_41658_MOESM1_ESM.docx]

# Preparation of ZnGa_2_O_4_ Nanoflowers and Their Full-color Luminescence Properties

# Yan Liu^1,^, Tingting Zheng^1,*^, Xiuyun Zhang^1,*^, Chen Chen^2,*^

1Department of Pharmacy, Shandong University of Traditional Chinese Medicine, Jinan, 250355, Shandong, China.

2Key Laboratory of New Material Research Institute, Department of Acupuncture-Moxibustion and Tuina, Shandong University of Traditional Chinese Medicine, Jinan ,250355, China.

*corresponding author: Tingting Zheng(ttz10_10@163.com), Xiuyun Zhang(zhangxiuyunsh@163.com) and Chen Chen(21129008@zju.edu.cn)

**Table S1** Coordination numbers (CN) of Zn^2+^, Ga^3+^, Mn^2+^ and Cr^3+^ and corresponding effective ion radius and the effective compensation factors (*φ*).

| Ions | CN = 4 | | CN = 6 | |
| --- | --- | --- | --- | --- |
|  | Ion radius (Å) | *φ* | Ion radius (Å) | *φ* |
| Zn^2+^ | 0.60 | 3.33 | / | / |
| Ga^3+^ | / | / | 0.62 | 4.83 |
| Mn^2+^ | 0.66 | 3.03 | 0.83 | 2.41 |
| Cr^3+^ | **/** | **/** | 0.62 | 4.83 |


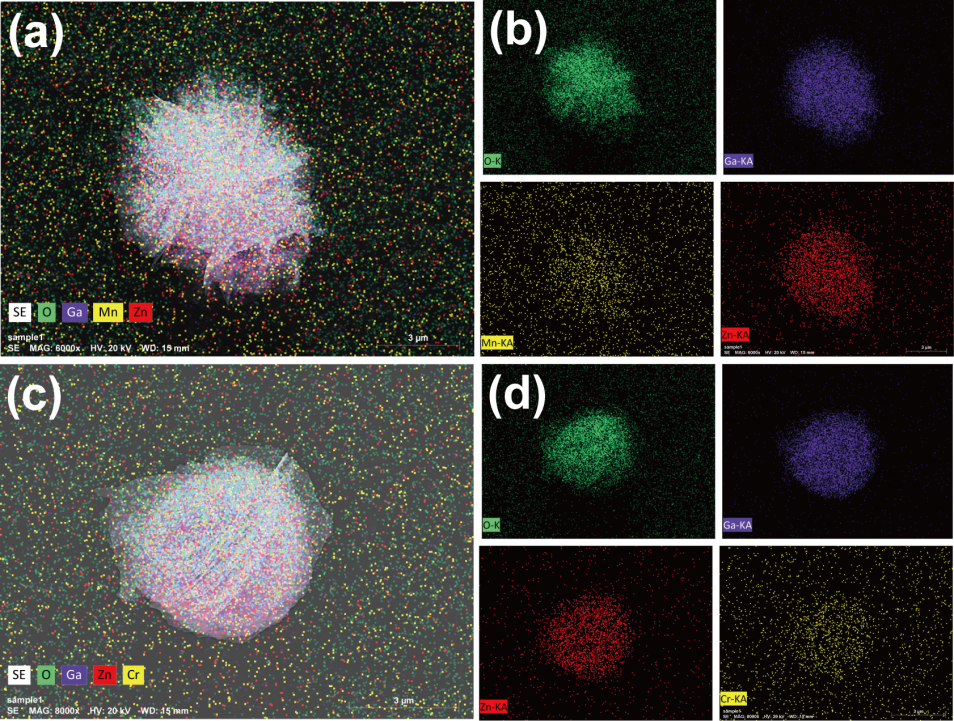


**Figure S1** Mapping of elements in (a)-(b) ZnGa_2_O_4_:Mn^2+^ and (c)-(d) ZnGa_2_O_4_:Cr^3+^.


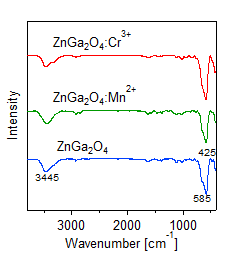


**Figure S2** The FT-IR spectra of ZnGa_2_O_4_, ZnGa_2_O_4_:Mn^2+^ and ZnGa_2_O_4_: Cr^3+^.

**Figure S3** The UV-Vis spectra of ZnGa_2_O_4_, ZnGa_2_O_4_:Mn^2+^ and ZnGa_2_O_4_: Cr^3+^.

**Figure S4** The enlarged photoluminescence excitation spectrum of ZnGa_2_O_4_:Mn^2+^ at 300-500 nm.

**Table S2** Fitting parameters obtained from bi-exponential fittings of PL dacays.

| Samples | *τ_1_* [ms] | *τ_2_* [ms] | *I_1_* | *I_2_* | *τ_avg_* [ms] |
| --- | --- | --- | --- | --- | --- |
| ZnGa_2_O_4_ | 0.13 | 1.47 | 77.76 | 18.02 | 1.10 |
| ZnGa_2_O_4_:Mn^2+^ | 1.70 | 8.59 | 22.24 | 47.35 | 8.00 |
| ZnGa_2_O_4_:Cr^3+^ | 0.97 | 6.73 | 31.84 | 18.90 | 5.60 |

*τ_avg_* is the average life span.
